# Thymic function and survival at advance ages in nursing home residents from Southern Italy

**DOI:** 10.1186/s12979-023-00340-0

**Published:** 2023-04-10

**Authors:** Ersilia Paparazzo, Silvana Geracitano, Vincenzo Lagani, Luigi Citrigno, Denise Bartolomeo, Mirella Aurora Aceto, Francesco Bruno, Raffaele Maletta, Giuseppe Passarino, Alberto Montesanto

**Affiliations:** 1 Department of Biology, Ecology and Earth Sciences, University of Calabria, Rende, 87036 Italy; 2 Biological and Environmental Sciences and Engineering Division (BESE), King Abdullah University of Science and Technology KAUST, Thuwal, 23952 Saudi Arabia; 3 SDAIA-KAUST Center of Excellence in Data Science and Artificial Intelligence, King Abdullah University of Science and Technology KAUST, Thuwal, 23952 Saudi Arabia; 4 Institute of Chemical Biology, Ilia State University, Tbilisi, 0162 Georgia; 5 National Research Council (CNR) - Institute for Biomedical Research and Innovation – (IRIB), 87050 Mangone, Cosenza, Italy; 6 Regional Neurogenetic Centre (CRN), Department of Primary Care, ASP Catanzaro, Lamezia Terme (CZ), 88046 Italy; 7 Association for Neurogenetic Research (ARN), Lamezia Terme (CZ), 88046 Italy

**Keywords:** Immunosenescence, Immunoageing, thymus, Advanced age, T-cell receptor rearrangement excision circles (sjTRECs), C-reactive protein (CRP), Mortality, Nursing home, Survival

## Abstract

**Background:**

Immunosenescence is a complex process characterized by an age-related remodelling of immune system. The prominent effects of the immunosenescence process is the thymic involution and, consequently, the decreased numbers and functions of T cells. Since thymic involution results in a collapse of the T-cell receptor (TCR) repertoire, a reliable biomarker of its activity is represented by the quantification of signal joint T-cell receptor rearrangement excision circles (sjTRECs) levels. Although it is reasonable to think that thymic function could play a crucial role on elderly survival, only a few studies investigated the relationship between an accurate measurement of human thymic function and survival at old ages.

**Methods and findings:**

By quantifying the amount sjTRECs by real-time polymerase chain reaction (PCR), the decrease in thymic output in 241 nursing home residents from Calabria (Southern Italy) was evaluated to investigate the relationship between thymic function and survival at old ages. We found that low sjTREC levels were associated with a significant increased risk of mortality at older ages. Nursing home residents with lower sjTREC exhibit a near 2-fold increase in mortality risk compared to those with sjTREC levels in a normal range.

**Conclusion:**

Thymic function failure is an independent predictor of mortality among elderly nursing home residents. sjTREC represents a biomarker of effective ageing as its blood levels could anticipate individuals at high risk of negative health outcomes. The identification of these subjects is crucial to manage older people’s immune function and resilience, such as, for instance, to plan more efficient vaccinal campaigns in older populations.

## Background

Ageing is a complex process characterized by a time-dependent functional decline that affects most living organisms. In humans several hallmarks have been identified and include genomic instability, telomere attrition, epigenetic alterations, loss of proteostasis, deregulated nutrient-sensing, mitochondrial dysfunction, cellular senescence, stem cell exhaustion, and altered intercellular communication [1]. The remodeling of the immune system that occurs with age, a process known as immunosenescence, is correlated to an increased vulnerability to infectious diseases, diminished responses to vaccination, and a susceptibility to age-related inflammatory diseases. It represents a multifactorial process caused by interactions among several components including genetic factors, gender, nutrition, physical exercise, and previous exposure to microorganisms [2–5]. Several data showed that the concentrations of some proinflammatory cytokines, chemokines, and adipokines are strong predictors of morbidity and mortality in elderly people [6, 7]. However, their analysis requires a special laboratory processing which can be a source of major errors in the diagnostic process. For example, the pre-analytical handling steps (e.g., blood collection tubes, specimen storage temperature and time, plasma or serum separation) in the analysis of cytokines can influence both sample quality and analytical results [8–11]. Therefore, appears necessary to search for new biomarkers capable of predicting survival at older age in an even more accurate way.

The prominent effects of the immunosenescence process are thymic involution and the consequently decreased numbers and functions of T cells, although also B cells are affected by this complex process [12]. Until some years ago, estimations on the timing of cessation of thymic function were imprecise and based on invasive and time-consuming expensive methods [13–17]. More recently, the possibility to monitor the thymic activity with an easy and efficient method has opened the opportunity to assess this biomarker in a cost-effective way [18]. In particular, since the age-related thymic involution results in a collapse of the T-cell receptor (TCR) repertoire, in humans the monitoring of this by-product of is a good candidate of thymic function. The signal joint T-cell receptor rearrangement excision circles (sjTRECs) are extra-chromosomal DNA by-products the rearrangements of gene segments encoding the variable parts of TCR α and β chains during intra-thymic development. sjTREC is a particular TREC arising through an intermediate rearrangement in the TCRD/A locus in developing TCRαβ + T lymphocytes [19]. sjTRECs-based methods, exploiting the observed age-related decline of sjTRECs content in human peripheral blood, show a relatively high prediction accuracy with the only limitation due to their tissue-specificity. In the last decade, it has been successfully used as a marker for age prediction from blood samples in forensic studies [19–21]. Although it is reasonable that thymic function could also have an important role on elderly survival, only a few studies investigated the relationship between an accurate measurement of human thymic function and survival at old ages [22–24]. In the present study, the decrease in thymic output in 241 nursing home residents from Calabria (Southern Italy) was evaluated to investigate the relationship between thymic function and survival at old ages.

## Results

General and clinical characteristics of the NH residents are reported in Table 1.

**Table 1:** General and clinical characteristics of the NH residents recruited in the present study. For continuous variables means and standard deviations (in parentheses) are reported. For categorical variables absolute frequencies and percentages (in parentheses) are reported

	All (N = 241)	Survivors (N = 191)	Dead (N = 50)	P-value
Age (years)	78.4 (7.59)	77.0 (7.19)	83.4 (6.96)	< 0.001
Sex, male (N,%)	83 (34.4%)	66 (34.6%)	17 (34.0%)	0.941
BMI (kg/m^2^)	27.2 (7.22)	28.2 (6.91)	22.9 (6.35)	< 0.001
Dependency in 1 or more ADL (N,%)	112 (46.5%)	68 (35.6%)	44 (88.0%)	< 0.001
ADD (N,%)	8 (3.3%)	5 (2.6%)	3 (6.0%)	0.456
UD (N,%)	53 (22.0%)	25 (13.1%)	28 (56.0%)	< 0.001
Depression (GDS > 5)* (N,%)	93 (47.4%)	78 (46.7%)	15 (51.7%)	0.617
Hypertension (N,%)	169 (70.1%)	128 (67.0%)	41 (82.0%)	0.059
Diabetes (N,%)	63 (26.1)	43 (22.5%)	20 (40.0%)	0.021
COPD (N,%)	50 (16.6%)	34 (17.8%)	16 (32.0%)	0.045
Heart failure (N,%)	18 (7.5%)	9 (4.7%)	9 (18.0%)	0.004
Ischemic heart disease (N,%)	48 (19.9%)	35 (18.3%)	13 (26.0%)	0.312
Stroke (N,%)	19 (7.9%)	6 (3.1%)	13 (26.0%)	< 0.001
CIRS-G score	12.5 (12.4)	8.8 (8.4)	26.0 (14.7)	< 0.001
Number of medications	6.8 (3.8)	6.3 (3.4)	8.8 (4.3)	< 0.001

BMI: body mass index; ADL: activities of daily living; GDS: geriatric depression scale; COPD: chronic obstructive pulmonary disease; ADD: Alzheimer’s disease dementia; UD: unspecified dementia; CIRS-G: cumulative illness rating score for geriatrics.

* 45 missing values

The mean age of these patients was 78.4 years, and around 34.4% of them were men. During the follow-up period (3 years), 50 NH residents (20.7%) died, while the remaining 191 survived (79.3%). About one half of enrolled patients had at least 1 ADL dependency at the time of admission. This percentage increased up to 88% in people who died during the follow-up period. Prevalence of diabetes, UD, COPD, heart failure, and stroke were significantly higher in NH residents who died during the follow-up, while there were no significant differences between the two groups (survivors vs. dead) for ischemic heart disease, hypertension, ADD, depression. The BMI was significantly higher in the survivors compared to the NH residents who died, whereas the latter used more medications than the survivor group.

### Age-related changes of sjTREC and CRP

**Fig. 1:**
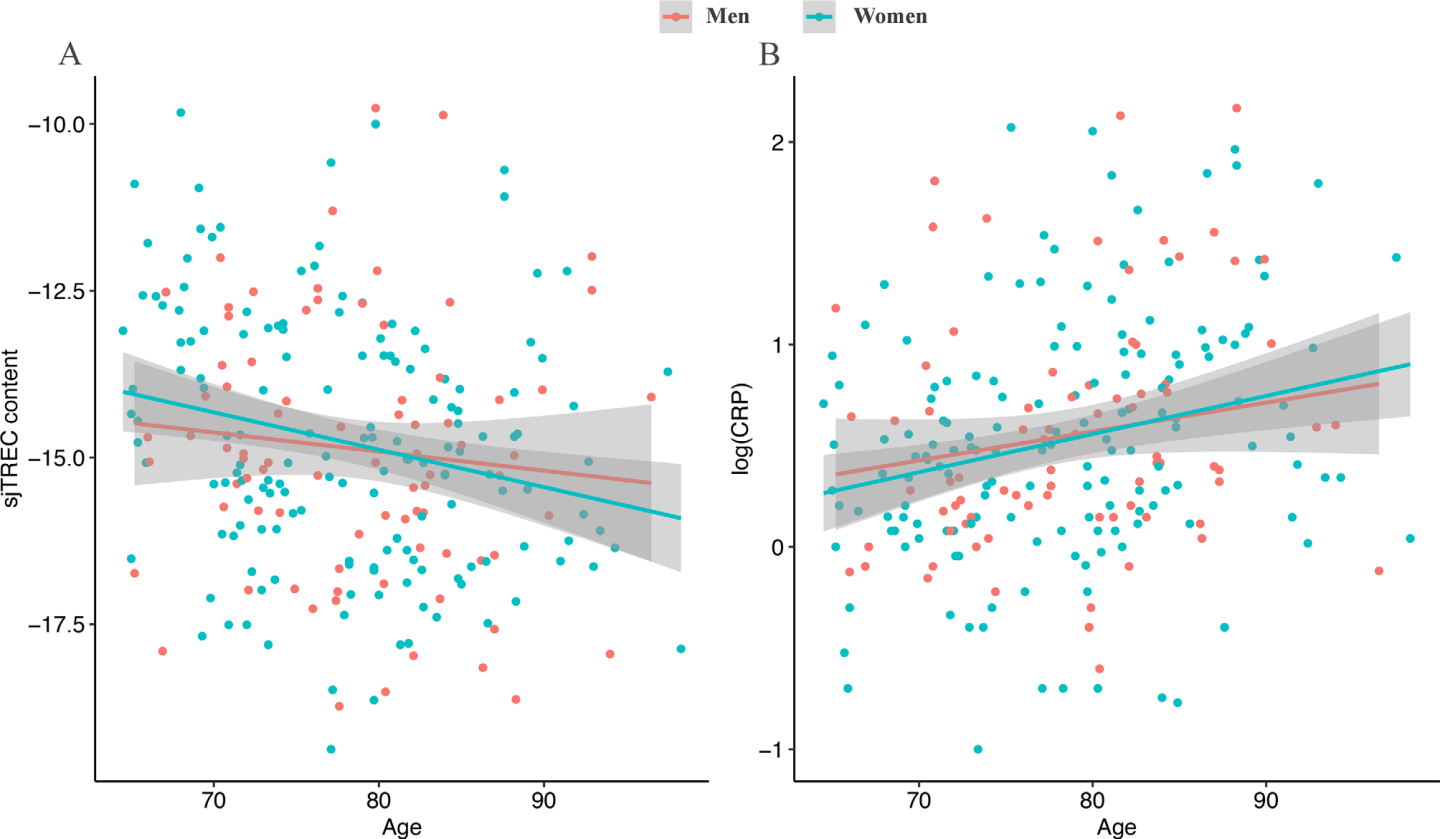
sjTREC (panel A) and log-transformed CRP (panel B) levels in 241 blood samples of different ages. In each panel, a regression line (in red for men and in green for women) to model the relationship between the immunosenescence marker and the age is reported

Figure 1 displays the age-related decline of sjTREC and CRP levels in the 241 analyzed blood samples. The sjTREC content exhibits a significant decline as a function of age (panel A, r=-0.187, P-value = 0.004), while CRP level (logarithmic scale) shows a significant age-related increase (panel B, r = 0.220, P-value < 0.001).

### Association between sjTREC and CRP levels and mortality

**Fig. 2:**
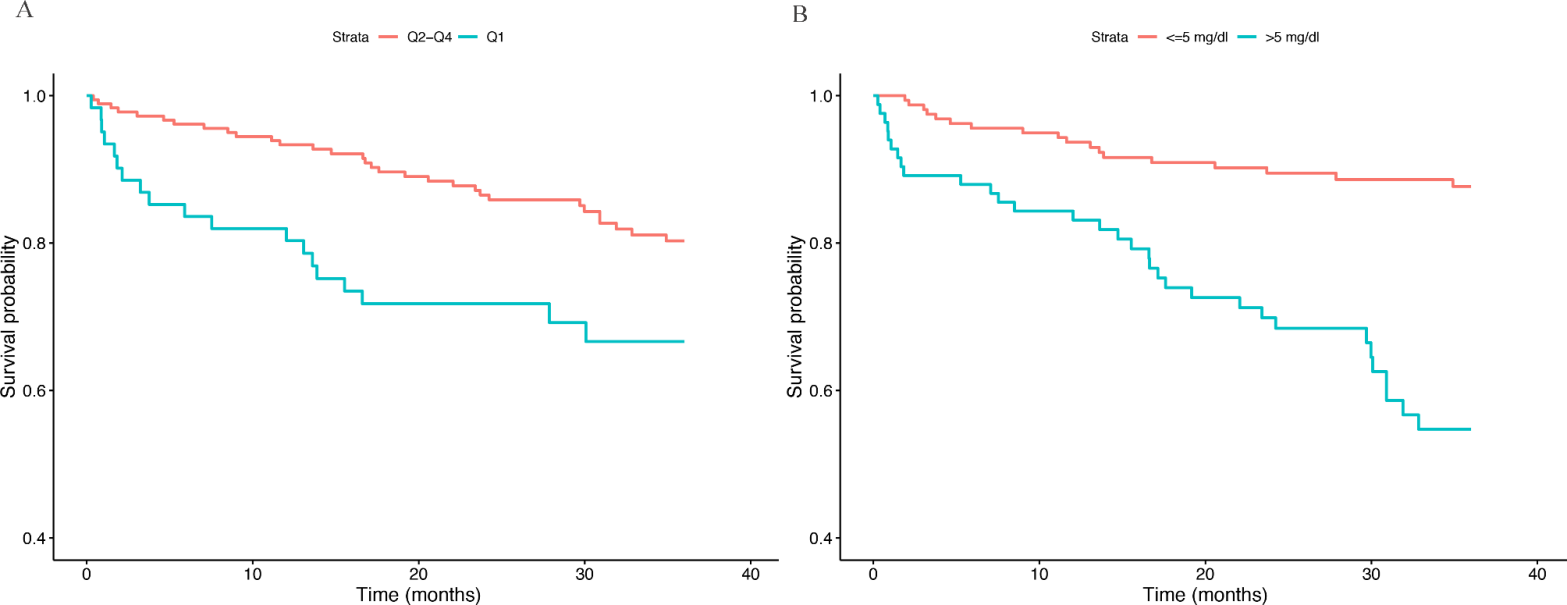
(A) Kaplan–Meier survival curves stratified according to the lowest quintile of sjTREC values. (B). Kaplan–Meier curves stratified by C-reactive protein according to the cutoff value of 5 mg/dl. For both parameters, survival curves for people at high risk of mortality (NH residents with sjTREC lower than − 16.25 units or with CRP level higher than 5 mg/dl) were reported in green, while the curves for people at low risk were reported in red

Kaplan–Meier survival curves according to the sjTREC content and CRP levels are reported in Fig. 2.

NH residents in the lowest sjTREC quartile (Q1< -16.25 units) exhibit the worst survival during the follow-up time (P-value = 0.007) (Fig. 2 panel A). NH residents with highest CRP levels (> 5 mg/dL) lived significantly shorter than subjects with normal CRP levels (P-value < 0.001) (Fig. 2 panel B).

Cox regression analyses confirmed these significant associations. In fact, NH residents with low sjTREC levels exhibit a more than 2-times higher risk of mortality (HR = 2.14, 95%CI = 1.21–3.80, P-value = 8.93*10^− 3^), while among the NH residents with highest CRP levels the mortality risk was around 4-times higher than residents with CRP in the normal range (HR = 4.09, 95%CI = 2.29–7.29, P-value = 1.91*10^− 6^). To check whether these immunosenescence markers had an independent effect on the mortality risk, we tested their effect in combination (model 1, unadjusted) and also adjusting for several potential confounders including age, gender, BMI, dementia, hypertension, diabetes, COPD, heart failure, stroke, comorbidity and number of medications (model 2 and 3, see Table 1). We found that sjTREC and CRP represent independent predictors of mortality risk and the estimated HR remained almost unchanged with respect the corresponding univariate estimates (HR = 2.34, 95%CI = 1.21–3.80, P-value = 0.004 for sjTREC; HR = 4.28, 95%CI = 2.39–7.64, P-value = 9.28*10^− 7^ for CRP) (Table 2). The age- and sex-adjusted model still confirmed these significant associations (model 2), but in model 3 the effect of sjTREC on the mortality risk still remained significant (HR = 2.48, 95%CI = 1.22–5.05, P-value = 0.010), while the effect of highest CRP levels became borderline (HR = 1.76, 95%CI = 0.88–3.52, P-value = 0.102). Other significant confounders associated with the outcome in model 3 were BMI (HR = 0.87, 95%CI = 0.79–0.96, P-value = 0.005), COPD (HR = 2.53, 95%CI = 1.15–5.56, P-value = 0.018), heart failure (HR = 2.91, 95%CI = 1.17–7.25, P-value = 0.019) and CIRS-G score (HR = 1.05, 95%CI = 1.02–1.08, P-value = 0.001). Also considered as a continuous, sjTREC content turned out to be an independent predictor of mortality (HR = 0.78; 95% CI 0.64–0.97, P-value = 0.022).

**Table 2:** Hazard Ratio for the relationship between sjTREC and CRP levels and survival chance during the follow-up time

	Model 1			Model 2			Model 3		
	HR	95%CI	P-value	HR	95%CI	P-value	HR	95%CI	P-value
SjTREC< -16.24	2.34	1.21–3.80	0.004	2.13	1.20–3.81	0.001	2.48	1.22–5.05	0.010
CRP > 5 mg/dl	4.28	2.39–7.64	< 0.001	3.69	2.05–6.65	< 0.001	1.76	0.88–3.52	0.102
Age	-	-	-	1.10	1.06–1.15	< 0.001	1.05	0.99–1.11	0.076
Sex	-	-	-	0.89	0.50–1.61	0.703	0.55	0.28–1.11	0.088
BMI							0.87	0.79–0.96	0.005
ADL					-	-	1.45	0.44–4.74	0.530
UD							1.01	0.43–2.36	0.985
Hypertension							2.18	0.86–5.55	0.096
Diabetes							1.28	0.61–2.68	0.507
COPD							2.53	1.15–5.56	0.018
Heart Failure							2.91	1.17–7.25	0.019
Stroke							1.80	0.85–3.80	0.116
CIRS-G					-	-	1.05	1.02–1.08	0.001
Number of medications							0.95	0.86–1.04	0.275

HR: Hazard Ratio; CI: Confidence Interval; BMI: body mass index; ADL: activities of daily living; COPD: chronic obstructive pulmonary; UD: unspecified dementia; CIRS-G: cumulative illness rating score for geriatrics.

## Discussion

Human ageing is a complex phenomenon characterized by a remodeling of the different organs, and systems, including the immune system [25]. In fact, the ageing process is accompanied by several important immune related changes, a phenomenon termed as immunosenescence one of the most prominent effects of which is represented by the thymic involution and the consequently decreased numbers and functions of T cells [12]. Imunosenescence plays a critical role in most chronic diseases in older people [26, 27] and it has been related with several comorbidities, such as cardiovascular risk [28, 29], cancer [30, 31], Alzheimer’s disease [27] or poor response to vaccines [32, 33]. Furthermore, the importance of immunosenescence is now receiving unprecedented emphasis during the COVID-19 pandemic, bringing to the fore the critical need to improve older people’s immune function and resilience [26]. Thus, the identification of a reliable biomarker of its activity might be useful to identify individuals at high risk, to evaluate more efficient strategies for immune rejuvenation and to plan effective campaigns of vaccination-mediated immunity in older individuals [34]. Among them, the measurement of biological markers such as naive T-cell subsets, thymulin level, and sjTREC count represent the more effective methods [35]. Since sjTREC quantification does not require viable cells, it is well-suited for assessing thymic function in large population samples in which the collected blood samples are appropriately stored. sjTRECs are episomal DNA circles in T-cells that are generated during excisional rearrangement of T-cell receptor genes. Although sjTREC echoes an outstanding biological aspect of the ageing process in humans, only a few studies investigated the relationship between an accurate measurement of human thymic function and survival at old ages [22, 23]. In this study, we demonstrated that thymic activity as assessed by sjTREC is a risk factor for mortality at advanced ages. In particular, we found that CRP raise and sjTREC reduction are independent markers of mortality among elderly institutionalized people. NH residents with low sjTREC levels exhibit a more than 2-times higher risk of mortality (HR = 2.14, 95%CI = 1.21–3.80, P-value = 8.93*10^− 3^), while among the NH residents with highest CRP levels the mortality risk was around 4-times higher than residents with CRP in the normal range (HR = 4.09, 95%CI = 2.29–7.29, P-value = 1.91*10^− 6^). However, while after adjusting for potential confounders such as age, BMI, chronic obstructive pulmonary disease, diabetes, heart failure and comorbidity, lower sjTREC levels still remained significantly associated with the mortality risk during the 3-year follow-up, the effect of highest CRP levels became borderline (Table 2, model 3).

In our cohort, thymic function failure was independently associated with all-cause mortality in the healthy elderly. While a number of studies investigated the relationship between CRP levels and mortality [36–39], only in a few reports the role of thymic function in human survival was investigated [22, 23]. In particular, we extended these preliminary results of these two studies by analysing a larger sample for a longer observational period with higher quality methodology (e.g., accidental deaths, are not discarded from our database) and taking into account other variables such as activity of daily living, depression, and several diseases assessed within the frame of a multidimensional evaluation.

The increased levels of CRP are also a marker of mortality in the healthy elderly, in a thymic-independent way [40], as also partially confirmed by the results of the present study. However, the sjTREC quantification does not require viable cells and is thus well-suited for assessing thymic function in appropriately stored blood samples and is therefore more useful than analyzing CRP levels.

### Limitations

The results reported here, despite being interesting for gerontology practice, present some limitations that should be considered. First, the sjTREC evaluation is restricted to blood samples and body parts containing blood and is not possible for other body parts or fluids, such as semen or saliva, that do not contain T cells in quantities required for sjTREC detection. In addition, a sufficient amount of DNA is required even in case of availability of these tissues. Further studies are therefore required to overcome this methodological limitation and to further validate this powerful biomarker. One further reason of concern is the disparity between survivors and deceased patients in terms of age, as well as in terms of presence of diabetes and other complications. This is a result of the retrospective nature of our study. We controlled for these potential confounders by including all measured ones in our multivariate models. Larger cohorts and different experimental designs may be needed in future studies for further confirming the validity of our findings. Lastly, the people who took part in this study all come from Calabria, a Southern Italian region known for having its own genetic peculiarities [41, 42]. The results we obtained should be confirmed in other cohorts of patients from different geographical areas.

## Conclusions

sjTREC represents a reliable biomarker of effective ageing whose blood levels could help to identify individuals at high risk of negative health outcomes. The identification of these subjects is crucial to plan more efficient vaccinal campaigns in older populations allowing to improve older people’s immune function and resilience. Further studies are needed to analyze the association between the levels of sjTREC and other well-established biomarkers of immunosenescence such as the positivity for cytomegalovirus (CMV) infection and the concentrations of some proinflammatory cytokines, chemokines, and adipokines.

## Methods

### Sample

Patients consecutively admitted to six participating nursing homes (NH) located within the province of Crotone and Cosenza (Calabria, Southern Italy) recruited between 2017 and 2018 were asked to participate in the study. A total of 258 NH residents aged 65 years and older were initially screened and enrolled. Exclusion criteria included any of these situations during the last 6 months: clinical data of active infections, hospital admission, antitumor therapy, or any treatment that could influence their immune status (e.g., corticosteroids). All recruited subjects underwent a multidimensional geriatric assessment with detailed clinical history, including anthropometric measures and a set of the most common tests to assess cognitive functioning, functional activity, physical performance, nutritional status and depression. In addition, a blood sample for DNA extraction was obtained at the same time of the geriatric assessment visit, when also blood for common clinical hematological tests was obtained. For the current analysis, NH residents with missing mortality data (n = 13) or missing values for C-reactive Protein (n = 4) were excluded, leaving 241 NH residents to be included in the analyses regarding sjTREC detection and survival analysis. An informed written consent was signed by all subjects or their legal representative. The study protocol was approved by the regional Ethical Committee, Catanzaro, Italy (Prot. CE 119/2016).

### Outcomes

Main outcome of the present study was to investigate the relationship between thymic function and overall mortality in NH residents. After the baseline visit, NH residents were followed-up every 12 months for 3 years to collect information about vital status. In the case of discharged patients, this information was collected by telephone call during which patients and/or their relative/caregiver were interviewed. For patients who died during the follow-up period, information about date, place, and cause of death was collected from death certificates provided by relatives or caregivers.

### Comprehensive geriatric assessment

The independence in activities of daily living (ADL, i.e. bathing, dressing, eating, independence in and out of bed) was assessed by using a modified version of an international and widely used scale, the Katz’ Index of activities of daily living [43]. The assessment was based on activities the subject was able to perform at the time of the visit. Each activity was scored as 0 if patients were unable to perform the task, and 1 for people able to perform such activity. Then, ADL scores ranged between 0 (unable to perform any activity) and 5 (able to perform all the activities). Depression was defined as having 15-item Geriatric Depression Scale (GDS) score > 5 [44]. Hypertension, heart failure (HF), diabetes, cancer, coronary artery disease (CAD), cerebrovascular diseases (CVD), chronic obstructive pulmonary disease (COPD), dementias (Alzheimer’s disease Dementia, ADD; unspecified dementia; UD) were also considered in the analyses. Overall comorbidity was assessed by Cumulative Illness Rating Score for Geriatrics (CIRS-G) [45]. CIRS-G evaluates the severity of coexisting diseases in 14 organ/systems scales, each ranging from 0 (problem absent) to 4 (severe problem with requirement of immediate treatment and/or severe organ/system failure). A sum score ranging from 0 to 56 points was then calculated by adding each system/organ score. In the case of multiple diseases affecting one organ/system, only the most severe condition was considered in the calculation of CIRS-G score.

### DNA samples preparation

Peripheral blood samples were collected in EDTA containing tubes from each human subject. Plasma/sera were used for routine laboratory analyses, while DNA was extracted from buffy coats following standard procedures. Genomic DNA was obtained by phenol/chloroform purification and then stored at − 20 °C until use. DNA concentration and purity were determined spectrophotometrically using NanoDrop™ One (Thermofisher).

### qPCR assays

Real-time PCR conditions and primers for quantification of *sjTREC* were carried out according to the previously published technique [46]. In brief, Real-time PCR experiments were carried out using a QuantStudio 3 Real-Time PCR System (Applied Biosystems) with a PowerUp SYBR Green Master mixture (Applied Biosystems). To quantify sjTREC levels, a relative quantification method was used with the TATA box Binding Protein (*TBP*) housekeeping gene as an internal reference (dCt = CtTBP − CtsjTREC). Real-time PCR was performed on approximately 50 ng DNA in 25 µL reaction volumes containing 500 nM of each primer. PCR conditions were 95 °C for 30 s, then 95 °C for 5 s, 60 °C for 15 s, and 72 °C for 20 s, for 45 cycles. The dissociation curve analysis was performed using default setting temperature. Amplicon size was 140 bp for sjTREC and 113 bp for TBP. All reactions were performed in triplicate and the average value from each sample was used for further data analysis.

### C-reactive protein quantification

C-reactive protein (CRP) serum levels were quantified with the CRPLX C-Reactive Protein (Latex) kit for the Cobas C 711 automated analyzer (Roche Diagnostics) according to the manufacturers’ instructions. Pathological CRP levels for adulthood, when using this kit, are established as CRP > 5 mg/l.

### Statistical analysis

Demographic, clinical characteristics, and outcomes data were summarized with counts and percentages for categorical variables, means (standard deviations) for normally distributed continuous variables, and medians (with interquartile ranges) for other continuous variables. The Kolmogorov–Smirnov test was used to check the normality of the related variables. Testing for independence in contingency tables was carried out by using the Fisher exact test. Kaplan–Meier estimates were used to obtain the survival curves with respect to the investigated immunosenescence markers (sjTREC and CRP). The obtained survival curves were then compared by log-rank test. Mortality risk of NH residents according to the immunosenescence markers also adjusting for potential confounders was compared by using Cox regression models. To control for the possible confounding effect of other variables on mortality, Hazard Ratios (HR) were adjusted for predictors that were significantly associated with mortality risk in the univariate analyses. The time from enrolment visit through the day of death was used as the time to failure variable for the model and NH survivors were censored on the day of the last follow-up visit. All statistical analyses were performed using R Statistical Software (version 4.1.0; R Foundation for Statistical Computing, Vienna, Austria).

## Data Availability

Research data generated that supports this research article will be shared upon request through a controlled access repository.

## List of abbreviations

ADD: Alzheimer’s disease Dementia
ADL: Activities of daily living
BMI: Body mass index
CAD: Coronary Artery Disease
CI: Confidence Interval
CIRS-G: Cumulative Illness Rating Scale for Geriatrics
CMV: Cytomegalovirus
COPD: Chronic obstructive pulmonary disease
COVID-19: COronaVIrus Disease 19
CRP: C-reactive protein
CVD: Cerebrovascular Diseases dementias
DNA: DeoxyriboNucleic Acid
GDS: Geriatric Depression Scale
HF: Heart Failure
HR: Hazard Ratios
NH: Nursing home
PCR: Polymerase chain reaction
sjTRECs: Signal joint T-cell receptor rearrangement excision circles
TBP: TATA box Binding Protein
TCR: T-cell receptor
UD: Unspecified Dementia
